# Challenges of Prostatic Tuberculosis: When Infection and Neoplasia Coexist

**DOI:** 10.7759/cureus.103976

**Published:** 2026-02-20

**Authors:** Karime Giselle Sandoval Enriquez, José Luis Parra Herrera, Melissa Carrillo Hernández, Manuel Angeles-Castellanos, José Angel Martínez Aguilar

**Affiliations:** 1 Department of Infectious Diseases, Universidad Cuauhtémoc Campus Aguascalientes, Aguascalientes, MEX; 2 Department of Infectious Diseases, Hospital General del Instituto de Seguridad y Servicios Sociales de los Trabajadores del Estado (ISSSTE), Aguascalientes, MEX; 3 Department of Internal Medicine, Hospital General del Instituto de Seguridad y Servicios Sociales de los Trabajadores del Estado (ISSSTE), Aguascalientes, MEX; 4 Department of Anatomical Sciences, Faculty of Medicine, Universidad Nacional Autonoma de Mexico, Mexico City, MEX; 5 Department of Internal Medicine, Hospital General Tercer Milenio, Aguascalientes, MEX

**Keywords:** extrapulmonary tuberculosis, genitourinary tuberculosis, granulomatous prostatitis, high-grade prostatic intraepithelial neoplasia, prostatic tuberculosis

## Abstract

Extrapulmonary tuberculosis (TB) accounts for 15%-20% of all TB cases, with genitourinary involvement being one of the less common forms. Prostatic TB is a rare entity that often presents nonspecific symptoms and is frequently diagnosed incidentally during histopathological examination.

We present the case of a 55-year-old man who sought care for persistent urinary symptoms, including dysuria, pollakiuria, straining, and bladder tenesmus. Laboratory studies showed elevated prostate-specific antigen levels. Ultrasound imaging demonstrated changes suggestive of chronic prostatitis. Subsequently, a histopathological study of a prostate biopsy with Ziehl-Neelsen staining reported the focal presence of acid-fast bacilli, a finding suggestive of prostatic TB.

Prostatic TB is a rare form of genitourinary TB that can clinically mimic benign processes such as benign prostatic hyperplasia or chronic urinary tract infections. Its diagnosis requires a high index of suspicion and histopathological confirmation. In this case, the pathological study was definitive.

This report highlights the importance of considering prostatic TB as a differential diagnosis in patients with chronic urinary symptoms and a poor response to conventional antibiotics, especially in areas endemic for TB.

## Introduction

Tuberculosis (TB) remains a major global public health concern. It's estimated that a quarter of the world's population is infected with Mycobacterium tuberculosis, with approximately 10 million new cases reported annually and 1.6 million deaths in 2021, positioning it as the second leading cause of mortality from infectious agents globally [1].

While the pulmonary form is the most common, extrapulmonary manifestations have shown an increase in recent decades, accounting for 15%-20% of all TB cases [2]. Of these, genitourinary involvement makes up approximately 10%-14% of cases, making it one of the most common extrapulmonary forms in some regions [3]. The prostate, however, is an unusual site of involvement; prostatic TB is considered an extremely rare entity, with an estimated frequency of just 2.6% of all genitourinary TB cases [4].

The diagnosis of prostatic TB presents a clinical challenge. The disease is often asymptomatic or presents with nonspecific symptoms that can mimic common conditions such as chronic prostatitis or even prostatic adenocarcinoma [3,5]. Serum prostate-specific antigen (PSA) levels can be elevated, further complicating the differential diagnosis with prostate cancer [5]. Histopathology is crucial for a definitive diagnosis; the presence of epithelioid granulomas with caseous necrosis and positive staining for acid-fast bacilli confirms the tuberculous etiology [6]. However, even this approach can be limited by the low sensitivity of traditional stains. In this context, molecular tests have gained a significant role. 

The importance of reporting cases of extrapulmonary TB lies in their rarity and the diagnostic challenge they pose. Unusual manifestations have been described, such as pancreatic TB [1], tuberculous tenosynovitis of the wrist [7], primary tuberculous pyomyositis of the forearm [8], and even central nervous system and cardiovascular involvement [9,10]. These reports expand the available literature and raise clinical awareness in scenarios where initial suspicion is typically low.

In this context, it is relevant to consider the interaction between TB and cancer. Various studies have documented that oncological patients have a higher risk of developing active TB, especially in scenarios of immunosuppression secondary to the neoplasm or chemotherapy treatment. In turn, TB can mimic or coexist with malignant processes, making the differential diagnosis difficult and delaying appropriate therapy. This bidirectional relationship is of particular interest in the genitourinary system, where granulomatous inflammation can mask or coexist with premalignant or malignant lesions, justifying a comprehensive clinicopathological approach [11].

## Case presentation

We report the case of a 55-year-old male. His relevant medical history included a three-month diagnosis of hypertension, with no known current treatment. He also had a previous COVID-19 infection and a history of chronic smoking for eight years at a rate of 20 cigarettes per day, resulting in a smoking index of 8 pack-years.

In March 2023, the patient presented dysuria, urinary frequency, straining, and bladder tenesmus. He was initially evaluated for prostatic enlargement and started on tamsulosin and finasteride, but with minimal improvement. Laboratory studies were ordered, which showed a total PSA of 66.3 ng/mL, with preserved renal and liver function. A complete blood count and urinalysis were within standard parameters. Subsequently, in June 2023, a urinary tract ultrasound was performed, which reported simple renal cysts (Bosniak I), Randall's plaques, and Grade I prostatic hyperplasia, with no evidence of a solid tumor.

Given the persistent elevation of PSA, the first transrectal prostate biopsy was performed in July 2023. The histopathological study reported fibroglandular hyperplasia and chronic prostatitis, with no evidence of malignancy.

In August 2023, the PSA was reported again as elevated at 79.5 ng/mL, with a free-to-total PSA ratio of 0.09 (9%), indicating a high risk of clinically significant prostate cancer.

Laboratory results at initial evaluation are summarized in Table 1. Reference ranges for total PSA and free-to-total PSA ratio were based on current urological guidelines [12].

**Table 1:** Laboratory results at initial evaluation. Reference ranges for total PSA and free-to-total PSA ratio were based on current urological guidelines [12]. PSA levels may vary according to age; however, values above 4.0 ng/mL are generally considered abnormal and warrant further clinical evaluation. PSA, prostate-specific antigen

Parameter	Patient value	Reference range	Interpretation
Total PSA	66.3 ng/mL	0-4.0 ng/mL	Markedly elevated
Total PSA (repeat)	79.5 ng/mL	0-4.0 ng/mL	Markedly elevated
Free-to-total PSA ratio	9%	>25% (low risk); <10% (high risk)	High-risk pattern

Subsequently, in May 2024, a prostate MRI was performed, which documented a volume of 31 cm³, a homogeneous peripheral zone with no suspicious lesions, and a heterogeneous transitional zone with no malignant-appearing nodules. The findings were classified as PI-RADS 2, consistent with inflammatory changes and prostatitis.

**Figure 1:**
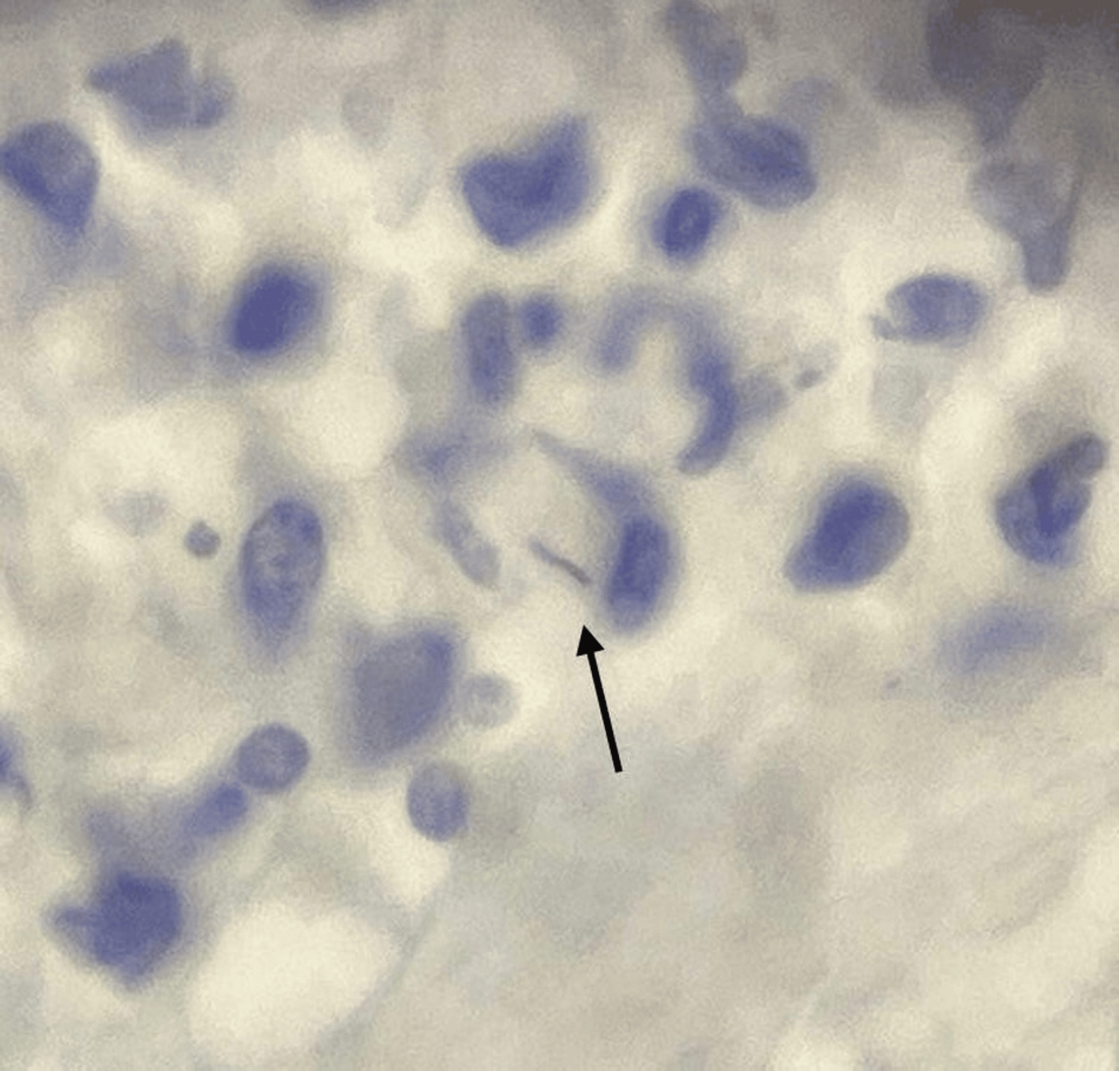
Chronic prostatitis with focal acid-fast bacilli stains positive for mycobacteria, suggestive of prostatic tuberculosis (arrow).

In August 2024, a second transrectal prostate biopsy was performed. The histopathological report evidenced chronic prostatitis, fibroglandular hyperplasia, basal cell hyperplasia, and the presence of focal high-grade prostatic intraepithelial neoplasia (HG-PIN). Additionally, a Ziehl-Neelsen stain was performed, which reported the focal presence of acid-fast bacilli, a finding suggestive of prostatic tuberculosis (Figure 1).

With these results, a diagnosis of prostatic tuberculosis was established, and treatment with standard anti-tuberculosis therapy (rifampicin, isoniazid, pyrazinamide, and ethambutol) was initiated for a total of six months, divided into an intensive phase and a maintenance phase. 

The patient's clinical condition improved, due to the histopathological report, the patient was referred to urology for the management of a precursor lesion (HG-PIN). A prostatectomy was offered; however, the patient declined. 

Currently, the patient remains under surveillance with a persistently elevated PSA and histopathological findings compatible with chronic prostatitis, fibroglandular hyperplasia, and HG-PIN, with no radiological evidence of invasive prostatic adenocarcinoma.

## Discussion

Prostatic TB is an uncommon condition within the spectrum of extrapulmonary TB. Although extrapulmonary TB accounts for up to 20% of global TB cases, prostatic involvement is rare, estimated at only 2.6% of genitourinary TB cases [6]. This low prevalence, coupled with its nonspecific clinical presentation and its ability to mimic adenocarcinoma, makes it a significant diagnostic challenge, especially in endemic areas.

Various studies have described up to six clinical presentations of prostatic TB, including: urinary obstructive symptoms, a clinical picture of chronic prostatitis, concomitant epididymal involvement, prostatic abscesses, infertility, and incidental findings in surgical specimens [13]. In the present case, the patient presented chronic prostatism, a persistently elevated PSA, and histopathological findings consistent with chronic prostatitis and granulomas, which reflect the nonspecificity of the initial symptoms.

The elevation of PSA in prostatic TB is particularly problematic. In most reports, PSA increases are moderate; however, there are documented cases with extreme elevations exceeding 1,700 ng/mL, which were initially interpreted as locally advanced adenocarcinoma [4]. This finding underscores that, while a marked elevation in PSA often suggests malignancy, granulomatous inflammation can produce an anomalous increase due to the destruction of glandular architecture and the release of the antigen [13]. In our patient, the PSA ranged from 66 to 79 ng/mL, with a reduced free-to-total ratio (9%), which reinforced the initial suspicion of clinically significant prostate cancer. The markedly elevated PSA levels (Table 1) further reinforced the initial suspicion of malignancy.

Imaging techniques also fail to establish a definitive diagnosis. Multiparametric magnetic resonance imaging may show hypointense lesions on T2, restricted diffusion, and patterns that mimic prostate cancer [10]. Some reports describe characteristic radiological patterns of prostatic TB, such as the *watermelon peel sign* in diffuse cases, but these are exceptional and not well-known findings [4]. In our case, the MRI reported inflammatory changes with a PI-RADS 2 classification, which, although suggesting a benign condition, did not explain the persistently elevated PSA values, leading to clinical uncertainty.

The definitive diagnosis is confirmed by histopathological examination. The presence of epithelioid granulomas with caseous necrosis and positive staining for acid-fast bacilli confirms the tuberculous etiology [6]. This observation has been corroborated in other extrapulmonary forms, such as hepatic TB, where the use of GeneXpert helped differentiate lesions that mimicked liver neoplasms or abscesses of other etiologies [14].

Literature reports also highlight the frequent confusion between prostatic TB and adenocarcinoma. Figueiredo et al. described multiple cases where granulomatous lesions were initially diagnosed as cancer [13]. Legesse et al. reported a patient with a PSA of 1,768 ng/mL, MRI findings compatible with PI-RADS 5, and suspicion of advanced cancer; however, histology showed isolated prostatic TB [4]. Similarly, even more unusual cases have been documented where prostatic TB and adenocarcinoma coexist in the same gland, which reinforces the need for representative and multiple biopsies to avoid missing either diagnosis [13].

To our knowledge, this is one of the few reported cases describing this association. It is particularly relevant as it documents the coexistence of prostatic TB with HG-PIN. HG-PIN is recognized as a precursor lesion for prostatic adenocarcinoma, with an increased risk of malignant progression. The literature on the simultaneous association of prostatic TB with premalignant lesions is scarce, making this finding an original contribution. This type of case raises questions about whether chronic granulomatous inflammation could promote proliferative changes in the prostatic epithelium or if it is a coincidental incidental finding. 

Finally, it is important to place the present case within the spectrum of extrapulmonary TB. Uncommon manifestations have been described, such as pancreatic TB [1], tuberculous pyomyositis [8], wrist tenosynovitis [7], hepatic TB [14], and head and neck TB [15]. All these rare forms share diagnostic difficulty, the ability to mimic neoplastic processes, and an almost absolute dependence on histopathology for confirmation. Added to this is the immunological perspective, which highlights how hematogenous dissemination and the inflammatory response condition the diversity of clinical presentations of extrapulmonary TB [16]. Furthermore, recent reviews have underlined that extrapulmonary TB remains a global diagnostic challenge, with epidemiological and terminological variations that explain its underreporting and frequent confusion with other pathologies [17].

## Conclusions

This case is particularly relevant, as it documents the presence of prostatic TB and its coexistence with HG-PIN. This association, rarely described in the literature, raises the possibility that chronic granulomatous inflammation may influence alterations in the prostatic epithelium and highlights the need for close monitoring due to the inherent oncological risk. Finally, prostatic TB should be considered in any patient with lower urinary tract symptoms, chronic prostatitis that is difficult to control, or an unexplained elevation of PSA, especially in endemic settings.
